# Clinical and Economic Outcomes in Patients With Alpha-1 Antitrypsin Deficiency in a US Medicare Advantage Population

**DOI:** 10.36469/001c.127446

**Published:** 2025-02-20

**Authors:** Nikhil Khandelwal, Jimmy Hinson, Trinh Nguyen, Alexjandro Daviano, Yihua Xu, Brandon T. Suehs, Sally Higgins, Marie Sanchirico, J. Michael Wells

**Affiliations:** 1 Takeda Pharmaceuticals USA, Inc., Lexington, Mass., USA; 2 Takeda Pharmaceuticals USA, Inc., Lexington, MA, USA; 3 External Healthcare Outcomes and Economic Research Humana Healthcare Research, Louisville, KY, USA; 4 Department of Medicine, Division of Pulmonary, Allergy, and Critical Care Medicine UAB Lung Health Center, University of Alabama at Birmingham, Birmingham, AL, USA

**Keywords:** alpha-1 antitrypsin deficiency, chronic obstructive pulmonary disease, Medicare Advantage population, healthcare resource utilization, rare diseases

## Abstract

**Background:**

Alpha-1 antitrypsin deficiency (AATD) testing rates and associated clinical and economic outcomes data in the US Medicare population are limited.

**Objective:**

To characterize individuals with AATD, describe clinical outcomes/healthcare research utilization (HCRU) among individuals with chronic obstructive pulmonary disease (COPD) with or without AATD, and identify AATD testing rates among individuals newly diagnosed with COPD.

**Methods:**

This retrospective, observational analysis of claims data included individuals from the Humana Research Database (aged 18-89 years) enrolled in Medicare Advantage Prescription Drug plans. Three cohorts included individuals with evidence of AATD; individuals with COPD + AATD matched to individuals with COPD; and individuals with newly diagnosed COPD. AATD health-related outcomes, such as pulmonary and extrapulmonary conditions or events, and economic outcomes, including inpatient admissions, emergency department visits, and physician visits, were examined independently during the pre-index and post-index periods and compared between those with ATTD and without AATD.

**Results:**

We identified 1103 individuals with AATD (aged 67.2 ± 10.0 years, 56.3% women, 94.5% White); overall, 22.2% had exacerbations, respiratory distress, and respiratory failure. Individuals with COPD and AATD (n = 742) were matched to individuals with COPD (n = 7420), based on age (68 ± 9 years), sex (55.0% women), and race (97.2% White). The AATD group had a higher proportion of emphysema (47.4% vs 18.7%), COPD exacerbations (40.6% vs 24.7%), and cirrhosis (4.0% vs 1.3%) than the non−AATD group. All-cause inpatient admissions (31.7% vs 27.3%), COPD-specific inpatient admissions (7.4% vs 4.3%), and COPD-specific emergency department visits (19.5% vs 10.8%) were higher in individuals who had ATTD than in those without AATD. AATD testing rates among individuals with newly diagnosed COPD increased slightly over time (2015: 1.07%; 2020: 1.49%). Individuals with COPD and AATD had more comorbidities and higher HCRU. Testing rates increased slightly but remained low.

**Discussion:**

Further research is needed to assess the impact of improved AATD testing on those with COPD.

**Conclusion:**

Increased awareness, earlier testing, and treatment may reduce the healthcare burden of AATD in the US Medicare population.

## INTRODUCTION

Alpha-1 antitrypsin deficiency (AATD) is a rare, inherited condition caused by mutations in the *SERPINA1* gene. Approximately 1:4500 to 1:2750 individuals worldwide are affected by these mutations, which lead to varying levels of deficiency in the circulating protease inhibitor alpha-1 antitrypsin (AAT). As a result, patients may develop lung and/or liver disease with varying severity.^1,2^ Pulmonary symptoms of AATD, including cough, dyspnea, and wheezing, intersect with those of common lung diseases such as asthma, chronic obstructive pulmonary disease (COPD), and emphysema.^3^ Similarly, chronic liver disease due to liver inflammation and resulting fibrosis leads to symptoms overlapping with those of cirrhosis, chronic viral hepatitis, or hepatocellular carcinoma.^4,5^ Converging symptoms with common liver and lung diseases, particularly COPD, make accurate and timely diagnosis of AATD challenging for healthcare professionals.^2^

AATD is observed in about 2% to 3% of patients in the United States with COPD.^6,7^ In 2019, COPD affected over 290 million people worldwide and was the third leading cause of death globally.^8,9^ Individuals over 60 years of age with COPD are less likely to have been tested for, or diagnosed with, AATD until its advanced stages and may have more severe clinical courses of the disease.^10,11^ While there are data showing that clinical burden and healthcare resource utilization (HCRU) are high for patients with AATD, particularly severe AATD, there is a paucity of evidence comparing the economic and clinical burden for patients with COPD and AATD compared with those with COPD alone.^12,13^ A previously conducted retrospective analysis of claims data in 711 patients with severe AATD who required hospitalization after AATD-related pulmonary events and 1963 patients with non-severe AATD reported that the mean annual incidences (mean) of emergency department (ED) (1.2 vs 0.4), inpatient (1.3 vs 0.1), and outpatient (10.3 vs 5.7) visits were higher in patients with severe AATD.^12^ Median annual costs were also higher for patients with severe AATD compared with non-severe AATD for ED ($185 vs $0), inpatient ($16 038 vs $0), and outpatient ($2663 vs $1114) visits.^12^ Another retrospective analysis of claims data matched AATD-associated COPD cases with up to 10 unique non-AATD-associated COPD controls and found that the incremental cost difference totaled $6861 and $5772 per patient before and after the index date.^13^ There is a need for real-world data to understand the clinical burden and HCRU in patients with COPD and AATD and to determine AATD testing rates in patients with COPD.

Furthermore, the American Thoracic Society, European Respiratory Society, American Association for Respiratory Care, and the Alpha-1 Foundation have made evidence-based recommendations on who should be screened for AATD.^7^ Despite these recommendations, real-world testing rates for AATD are low, and AATD remains substantially underdiagnosed.^4,14^ Epidemiological surveys estimate that less than 10% of individuals with AATD in the United States have been identified.^4,15,16^

The Medicare Advantage Prescription Drug (MAPD) plan population consists predominantly of age-eligible older adults, but it also includes younger adults with certain disabilities and special needs.^17^ As such, this population is suitable to study real-world healthcare trends including AATD testing, clinical outcomes, and economic burden of patients with COPD, both with and without AATD. In this study, we aimed to: (1) characterize individuals diagnosed with AATD based on demographic and clinical characteristics (cohort 1); (2) describe clinical outcomes, HCRU, and spending outcomes among individuals with COPD with evidence of AATD and compare them with a matched COPD cohort without AATD (cohort 2); and (3) identify the proportion of individuals newly diagnosed with COPD who received testing for AATD (cohort 3).

## METHODS

### Identification of Individuals With AATD (Cohort 1) or Newly Diagnosed COPD (Cohort 3)

**Figure 1.**
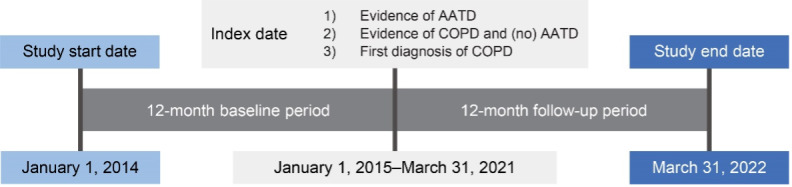
Study Design Abbreviations: AATD, alpha-1 antitrypsin deficiency; COPD, chronic obstructive pulmonary disease. Individuals with (1) evidence of AATD; (2) evidence of COPD with or without AATD; (3) first diagnosis of COPD with index date between January 1, 2015, and March 31, 2021, were identified. All eligible patients had available claims data for the 12-month pre-index and post-index period.

**Figure 2.**
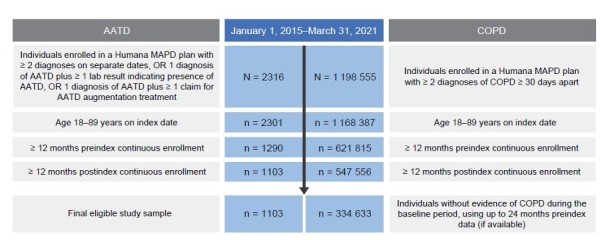
Identified Individuals With COPD or AATD Based on Inclusion and Exclusion Criteria Abbreviations: AATD, alpha-1 antitrypsin deficiency; COPD, chronic obstructive pulmonary disease.

This retrospective, observational study used administrative claims data collected between January 1, 2014, and March 31, 2022, to identify individuals with AATD (cohort 1) and individuals with newly diagnosed COPD (cohort 3) (**Figure 1**). Claims data used in this study were from fully insured commercial and Medicare members in Humana’s Research Database and included enrollment information (demographics, coverage start and end dates), medical and pharmacy claims, and laboratory results. Currently, 5.4 million members are in the Humana Medicare Advantage program as of December 31, 2023. The database includes administrative claims data for approximately 30 million individuals enrolled from 2007 to the present. The Humana Healthcare Research Human Subject Protection Office determined that this research does not constitute human subjects research in compliance with applicable laws, ethical standards, and Humana policies; therefore, it is exempt from IRB review. Eligible individuals were aged 18 to 89 years, enrolled in an MAPD plan, and diagnosed with AATD or COPD between January 1, 2015, and March 31, 2021 (**Figure 1; Figure 2**). Patients with COPD had no evidence of a diagnosis or positive lab test for AATD or augmentation treatment at any time during the study period. *International Classification of Diseases–Ninth/Tenth Revision–Clinical Modification* diagnosis codes, Current Procedure Terminology codes, National Drug Codes, and Generic Product Identifier codes were used to identify diagnoses, procedures, and medication utilization in the claims database. Patient data were available for at least 12 months pre-index date (ID) and post-ID (**Figure 1; Figure 2**). Among cohort 3, individuals with evidence of COPD during the pre-index baseline period were excluded. For cohort 2, patients with unknown race or race other than White or Black were excluded. Patients were excluded from all 3 cohorts if they had less than 12 months pre-index or post-index of continuous enrollment.

### Study Variables and Analyses

For all 3 cohorts, we described baseline patient demographics and clinical characteristics, including Deyo-Charlson Comorbidity Index (DCCI) scores and Elixhauser Comorbidity Index (ECI)–related comorbidities, measured during the 12-month pre-index date period. AATD severity in cohort 1 was defined based on (1) AAT laboratory values less than 50 mg/dL any time during the study period, or (2) an AATD diagnosis and hospitalization event for specific clinical conditions during the ID period, or (3) an AATD diagnosis during the ID period and baseline use of supplemental oxygen. During the 12-month pre-index and post-index periods, we measured AATD testing in individuals with newly diagnosed COPD (cohort 3). AATD-related health and economic outcomes were analyzed in individuals with COPD and AATD compared with a matched cohort of individuals with COPD and without AATD (cohort 2) during the pre-index and post-index periods (see **Supplemental Table S1** for detailed descriptions of study variables and analyses).

**Cohort matching (cohort 2):** Individuals with COPD and AATD were matched to individuals with COPD without AATD using a risk-set matching approach, which included age, sex, and race (White and Black patients only). Individuals with COPD and AATD were defined similarly as those with AATD in cohort 1 but also had at least 1 observed diagnosis of COPD during the 12 months prior to the index AATD diagnosis. Individuals with COPD and without AATD were identified from each calendar year based on at least 1 observed COPD diagnosis code, with the first diagnosis date as the index date. As with the COPD with AATD cohort, the COPD without AATD cohort also required patients to have at least 1 other COPD diagnosis during the 12-month pre-index period. Patients with evidence of AATD any time during the study period were excluded from the COPD-without-AATD group. Patients with unknown race or race other than White or Black were excluded from both groups. Matching was completed within each calendar year independently. Control individuals already matched in previous years were not eligible for matching in later years (see **Supplemental Figure S1** for procedure summary).

**Statistical analysis**: The baseline demographics and clinical characteristics were reported with descriptive statistics including mean, standard deviation, median, and interquartile range (IQR) for continuous variables, and counts and percentages for categorical variables. Bivariate statistical tests were performed to compare measures for individuals with evidence of severe AATD with individuals without evidence of severe AATD (cohort 1). Bivariate comparisons of baseline characteristics between individuals with COPD with and without AATD were conducted (cohort 2). AATD health-related outcomes, such as pulmonary and extrapulmonary conditions or events, and economic outcomes, including inpatient admissions, ED visits, and physician visits, and hospitalizations due to COPD exacerbations in the primary diagnosis position or any diagnosis position, were examined independently during the pre-index and post-index periods and compared between the AATD cases and control groups. *P* values were calculated using the χ^2^ test for categorical variables and Wilcoxon rank sum test for continuous variables (*P* values are reported in **Supplemental Tables S2-S8**).

## RESULTS

### Characterization of Individuals With AATD (Cohort 1)

Analysis of claims records of US individuals enrolled in a Humana MAPD plan between January 1, 2015, and March 31, 2021, identified 1103 individuals with evidence of AATD (**Figure 2**). Overall, 71.6% (n = 790) of individuals had no evidence of severe AATD and 28.4% (n = 313) of individuals showed evidence of severe AATD. Individuals with severe AATD were younger (65.7 [±10] years) than individuals with no evidence of AATD (67.8 [±10] years) (*P* < .001), and most patients across AATD categories were identified as White (94% to 98%) (**Table 1**).

**Table 1.** Demographics and Clinical Characteristics for Individuals With AATD and Those With or Without Evidence of Severe AATD

**Measures**	**All AATD**	**Severe AATD^a^**	**No Evidence of Severe AATD**
n	1103	313	790
Age, years			
Mean (SD)	67.2 (10.0)	65.7 (10.0)	67.8 (9.9)
Median (IQR)	69.0 (61.0-74.0)	67.0 (59.0-3.0)	69.0 (62.0-75.0)
Sex, n (%)			
Men	482 (43.7)	136 (43.5)	346 (43.8)
Women	621 (56.3)	177 (56.5)	444 (56.2)
Race, n (%)			
White	1042 (94.5)	294 (93.9)	748 (94.7)
Black	28 (2.5)	11 (3.5)	17 (2.2)
Other	16 (1.5)	<10	14 (1.8)
Unknown	17 (1.5)	<10	11 (1.4)
Geographic region, n (%)			
Northeast	30 (2.7)	<10	25 (3.2)
Midwest	235 (21.3)	67 (21.4)	168 (21.3)
South	726 (65.8)	210 (67.1)	516 (65.3)
West	112 (10.2)	31 (9.9)	81 (10.3)
Population density, n (%)			
Urban	575 (52.5)	164 (52.9)	411 (52.3)
Suburban	368 (33.6)	109 (35.2)	259 (33.0)
Rural	146 (13.3)	34 (11.0)	112 (14.2)
Unknown	<10	<10	<10
LIS, n (%)	251 (22.8)	89 (28.4)	162 (20.5)
DE, n (%)	102 (9.2)	34 (10.9)	68 (8.6)
LIS or DE, n (%)	254 (23.0)	89 (28.4)	165 (20.9)
Deyo-Charlson Comorbidity Index			
Mean (SD)	2 (2)	3 (2)	2 (2)
Median (IQR)	1 (1-3)	2 (1-4)	1 (1-3)
Elixhauser Comorbidity Index			
Mean (SD)	4 (3)	5 (3)	3 (2)
Median (IQR)	3 (2-5)	4 (2-7)	3 (1-4)

Abbreviations: AAT, alpha-1 antitrypsin; AATD, alpha-1 antitrypsin deficiency; DE, dual eligible; ID, index date; IQR, interquartile range; LIS, low-income subsidy; SD, standard deviation.

^a^Severe AATD was defined as AAT laboratory values <50 mg/dL any time during study period or AATD diagnosis and a hospitalization event for specific clinical conditions during the ID period or AATD diagnosis during ID period and baseline use of supplemental oxygen.

ID period: January 1, 2015–March 31, 2021.

During the pre-index period, individuals with AATD had a high burden of pulmonary and extrapulmonary comorbidities (**Supplemental Figure S2**). Among individuals with severe AATD, a majority (91.7%) had been diagnosed with various respiratory diseases such as COPD, emphysema, and moderate-to-severe asthma. Overall, 22.2% of individuals with AATD had exacerbations, respiratory distress, and respiratory failure. Extrapulmonary manifestations of AATD such as panniculitis, chronic hepatitis, chronic kidney disease, or congestive heart failure were observed in 78.9% of individuals with severe AATD.

Clinical characteristics, HCRU, and spending in individuals with COPD and AATD were compared with that in individuals with COPD alone (cohort 2)

### Demographics and Clinical Characteristics

To compare clinical characteristics, HCRU, and spending of individuals with COPD and AATD with individuals with COPD alone, we matched 2 patient cohorts. A total of 742 individuals with COPD and evidence of AATD were matched to 7420 individuals with COPD and no evidence of AATD based on age, sex, and race. This cohort had a mean age of 68 (±9) years; 55.0% were women, 97.2% were White, and approximately 25% to 28% of individuals in both groups had used the Medicare Part D low-income subsidy (LIS) (**Supplemental Table S6)**.

The baseline mean DCCI was similar among individuals with evidence of AATD and those without evidence of AATD (2.3 vs 2.5; *P* = .07). However, more individuals with evidence of AATD had a DCCI score of at least 1 compared with individuals without evidence of AATD (93.9% vs 88.8%, *P* < .001). The overall ECI was balanced between the 2 groups (standardized difference = 0.06); however, there were a few notable differences in ECI-related comorbidities among individuals with and without evidence of AATD. Individuals with AATD had a significantly higher prevalence of liver failure (6.5% vs 4.5%, *P* = .018) and weight loss (5.8% vs 3.8%; *P* = .008) than those without AATD (**Supplemental Figure S3**; see full list of comorbidities in **Supplemental Table S6**). Individuals without AATD had a significantly higher prevalence of peripheral vascular disease (20.1% vs 15.2%, *P* = .002), obesity (16.3% vs 13.1%, *P* = .024), and substance abuse disorder (6.0% vs 3.8%; *P* = .014) than individuals with AATD.

When comparing individuals with COPD with evidence of AATD vs those without evidence of AATD during the pre-index period, significantly higher rates of emphysema (47.4% vs 18.7%, *P* < .001) and COPD exacerbations (40.6% vs 24.7%, *P* < .001) were observed. The same trends remained during the post-index period (emphysema: 53.1% vs 24.2%, *P* < .001; COPD exacerbations: 41.6% vs 30.4%, *P* < .001) (**Table 2**). Among extrapulmonary events, individuals with COPD and evidence of AATD experienced significantly higher rates of cirrhosis during the pre-index period (4.0% vs 1.3%, *P* < .001) and post-index period (4.3% vs 1.7%, *P* < .001) than individuals without evidence of AATD. Conversely, individuals without evidence of AATD experienced significantly higher rates of diabetes in the pre-index period (36.5% vs 28.7%, *P* < .001) and post-index period (38.0% vs 29.9%, *P* < .001) than those with evidence of AATD.

**Table 2.** Pre-index and Post-index Pulmonary and Extrapulmonary Events in Individuals With COPD and With or Without AATD

**Measures**	**Pre-index, n (%)**		**Post-index, n (%)**	
	**Individuals With COPD and No AATD (n = 7420)**	**Individuals With COPD and AATD (n = 742)**	**Individuals With COPD and No AATD (n = 7420)**	**Individuals With COPD and AATD (n = 742)**
Pulmonary conditions				
Bronchitis, not specific	1020 (13.7)	124 (16.8)	1145 (15.4)	135 (18.2)
Bronchitis (chronic + not specific)	1294 (17.4)	154 (20.8)	1475 (19.9)	168 (22.6)
Bronchiectasis	192 (2.6)	66 (8.9)	226 (3.0)	85 (11.5)
Emphysema	1390 (18.7)	352 (47.4)	1798 (24.2)	394 (53.1)
Adult-onset asthma	1078 (14.5)	164 (22.1)	1083 (14.6)	152 (20.5)
Events				
Exacerbations of COPD	1834 (24.7)	301 (40.6)	2253 (30.4)	309 (41.6)
Pneumothorax	80 (1.1)	18 (2.4)	86 (1.2)	14 (1.9)
Procedures				
Lung transplant	<10	16 (2.2)	<10	2.40%
Extrapulmonary				
Cirrhosis	99 (1.3)	30 (4.0)	126 (1.7)	32 (4.3)
Diabetes	2710 (36.5)	213 (28.7)	2822 (38.0)	222 (29.9)
Eosinophilia	41 (0.6)	<10	84 (1.1)	17 (2.3)
Hepatocellular carcinoma	<10	<10	<10	<10

Abbreviations: AATD, alpha-1 antitrypsin deficiency; COPD, chronic obstructive pulmonary disease.

Post-index hospitalizations due to COPD exacerbations were significantly higher among individuals with AATD than those without AATD for the primary diagnosis position (6.3% vs 3.4%; *P* < .001) or any diagnosis position (15.0% vs 8.8%; *P* < .001) (**Supplemental Table S7**). When any diagnosis position was used, there were also significantly higher incidences of emphysema (3.2% vs 1.3%, *P* < .001) among those with AATD compared with individuals without AATD (**Supplemental Table S7)**.

### HCRU and Spending

HCRU was higher among individuals with COPD and evidence of AATD than among those without evidence of AATD for rates of inpatient admissions (31.7% vs 27.3%, *P* = .011) and physician office visits (99.7% vs 98.9%, *P* = .032) (**Table 3)**. Individuals with COPD and evidence of AATD had higher rates of COPD-specific inpatient admissions (7.4% vs 4.3%, *P* < .001) and ED visits (19.5% vs 10.8%, *P* < .001) than the non-AATD group, but there was no significant difference in the proportion of patients with all-cause ED visits (41.8% vs 40.6%, *P* = .521). The COPD-specific average length of stay was similar in the AATD group compared with the non-AATD group (mean, 6.3 vs 5.4 days, *P* = .230; median [IQR], 7 [4-14] vs 7 [4-13] days).

**Table 3.** Post-index HCRU in Individuals With COPD and With or Without AATD

**Measures^a^**	**Individuals With COPD Without AATD (n = 7420)**	**Individuals With COPD With AATD (n = 742)**
Inpatient admissions		
All-cause		
With an IP visit, n (%)	2025 (27.3)	235 (31.7)
Length of stay, mean (SD)	11.6 (15.8)	10.8 (10.6)
Length of stay, median (IQR)	7 (4–13)	7 (4–14)
COPD-specific^b^		
With an IP visit, n (%)	321 (4.3)	55 (7.4)
Length of stay, mean (SD)	5.4 (7.1)	6.3 (5.7)
Length of stay, median (IQR)	4 (2–6)	4 (3–7)
Pulmonary event-specific^c^		
With an IP visit, n (%)	24 (0.3)	<10
Length of stay, mean (SD)	3.9 (4.6)	4.0 (1.0)
Length of stay, median (IQR)	2 (1–4)	4 (3–5)
ED visits		
All-cause, n (%)	3010 (40.6)	310 (41.8)
ED visits, mean (SD)	3.1 (3.5)	2.9 (3.4)
ED visits, median (IQR)	2 (1–4)	2 (1–4)
COPD-specific,^b^ n (%)	799 (10.8)	145 (19.5)
ED visits, mean (SD)	1.8 (1.6)	1.9 (1.9)
ED visits, median (IQR)	1 (1–2)	1 (1–2)
Pulmonary event-specific,^c^ n (%)	63 (0.8)	15 (2.0)
ED visits, mean (SD)	1.3 (0.7)	1.7 (2.0)
ED visits, median (IQR)	1 (1–1)	1 (1–1)
Physician office visits		
All-cause, n (%)	7338 (98.9)	740 (99.7)
Office visits, mean (SD)	11.5 (8.1)	13.4 (7.9)
Office visits, median (IQR)	10 (6–15)	12 (8–17)

Abbreviations: AATD, alpha-1 antitrypsin deficiency; COPD, chronic obstructive pulmonary disease; ED, emergency department; HCRU, healthcare resource utilization; IQR, interquartile range; IP, inpatient; SD, standard deviation.

^a^Spending and HCRU are measured using data from the 12-month post-index period.

^b^COPD-specific inpatient and ED visits were identified based on medical claims with primary diagnosis of COPD or exacerbation.

^c^Pulmonary event–related utilizations were calculated based on medical claims with primary diagnosis of the following conditions: lung transplant, pulmonary circulatory disorder, tuberculosis, panniculitis, vasculitis, aneurysms, fibromuscular dysplasia, granulomatosis with polyangiitis, eosinophilia, and adult-onset asthma.

Median all-cause healthcare spending in individuals with COPD and evidence of AATD were significantly higher at 12-month follow-up than in individuals with COPD and no evidence of AATD ($19 375 vs $11 113, *P* < .001) (**Supplemental Table S8).** Additionally, individuals with COPD and AATD had significantly higher median all-cause medical spending ($8492 vs $6230, *P* < .001), COPD-specific medical spending ($815 vs $219, *P* < .001), and all-cause pharmacy spending ($5564 vs $2521, *P* < .001) compared with individuals with COPD and no AATD.

### Characterization of Individuals With COPD and AATD Testing Rates (Cohort 3)

Analysis of claims records identified 334 633 individuals newly diagnosed with COPD (**Figure 2**). Real-world testing rates were found to be low for AATD among US individuals newly diagnosed with COPD. Among the 334 633 individuals newly diagnosed with COPD, 1.04% had evidence of AATD testing in their claims records (**Table 4**). Testing rates for AATD were higher among younger, White, and female individuals with COPD living in the northeastern United States. Suburban areas had the highest testing rates for AATD (1.11%) followed by urban areas (1.02%); rural areas had the lowest testing rates (0.98%). Testing rates for AATD among individuals newly diagnosed with COPD increased slightly over time, ranging from 1.07% in 2015 to 1.49% in 2020 (**Supplemental Figure S4**).

**Table 4.** Demographics and Clinical Characteristics for Individuals With Newly Diagnosed COPD, Those With COPD Tested for AATD, and Those With COPD Not Tested for AATD

**Measures**	**Newly Diagnosed Individuals With COPD (n = 334 633)**	**Individuals With COPD Tested for AATD (n = 3470)**	**Individuals With COPD Not Tested for AATD (n = 331 163)**
Age, y			
Mean (SD)	70.7 (9.0)	67.9 (9.1)	70.7 (9.0)
Median (IQR)	71.0 (66.0–77.0)	69.0 (63.0–74.0)	71.0 (66.0–7.07)
Sex, n (%)			
Men	158 034 (47.2)	1507 (43.4)	156 527 (47.3)
Women	176 595 (52.8)	1963 (56.6)	174 632 (52.7)
Race, n (%)			
White	273 892 (81.8)	2955 (85.2)	270 937 (81.8)
Black	45 990 (13.7)	344 (9.9)	45 646 (13.8)
Other	11 401 (3.4)	122 (3.5)	11 279 (3.4)
Unknown	3350 (1.0)	49 (1.4)	3 301 (1.0)
Geographic region, n (%)			
Northeast	8794 (2.6)	108 (3.1)	8686 (2.6)
Midwest	66 719 (19.9)	706 (20.3)	66 013 (19.9)
South	224 638 (67.1)	2294 (66.1)	222 344 (67.1)
West	34 482 (10.3)	362 (10.4)	34 120 (10.3)
Population density, n (%)			
Urban	192 673 (58.4)	1961 (57.2)	190 712 (58.4)
Suburban	91 504 (27.7)	1017 (29.7)	90 487 (27.7)
Rural	43 290 (13.1)	424 (12.4)	42 866 (13.1)
Unknown	2339 (0.7)	28 (0.8)	2311 (0.7)
LIS, n (%)	72 081 (21.5)	763 (22.0)	71 318 (21.5)
DE, n (%)	26 398 (7.9)	276 (8.0)	26 122 (7.9)
LIS or DE	72 814 (21.8)	768 (22.1)	72 046 (21.8)
Deyo-Charlson Comorbidity Index			
Mean (SD)	1.6 (1.9)	1.9 (2.1)	1.6 (1.9)
Median (IQR)	1.0 (0.0–2.0)	1.0 (0.0–3.0)	1.0 (0.0–2.0)
Elixhauser Comorbidity Index			
Mean (SD)	3.0 (2.7)	3.5 (2.9)	2.9 (2.7)
Median (IQR)	2.0 (1.0–4.0)	3.0 (1.0–5.0)	2.0 (1.0–4.0)

Abbreviations: AATD, alpha-1 antitrypsin deficiency; COPD, chronic obstructive pulmonary disease; DE, dual eligible; IQR, interquartile range; LIS, low-income subsidy; SD, standard deviation.

There was a higher burden of specific comorbidities among individuals with COPD who were tested for AATD compared with those not tested for AATD (DCCI: mean 1.9 vs 1.6 and median 1.0 vs 1.0; ECI: mean 3.5 vs 2.9 and median 2.0 vs 3.0) (**Table 4**). Individuals with COPD and with specific comorbid conditions such as anxiety (25.2% tested vs 19.5% untested, *P* < .001), obesity (16.4% tested vs 12.5% untested), or liver failure (19.7% tested vs 3.0% untested, *P* < .001) were more likely to be tested for AATD than not tested (**Supplemental Figure S5**).

## DISCUSSION

Individuals with severe AATD are younger and have more comorbidities than those with non-severe AATD (objective 1, cohort 1)

In this study, we characterized individuals with evidence of AATD using the MAPD population. Enrolled individuals were predominantly age-eligible older adults, but younger adults with certain disabilities and special needs were also enrolled. As such, this population was suitable to study real-world data of patients with chronic respiratory diseases such as COPD and AATD. We found that individuals with severe AATD were younger than those with non-severe AATD, possibly because younger individuals experiencing comorbidities might receive AATD testing more readily compared with older patients. Overall, the prevalence of pulmonary, cardiac, and renal comorbidities was high among individuals with AATD and even higher among individuals with severe AATD. Prior studies reported similar outcomes for pulmonary and cardiac comorbidities.^12,18^

Individuals with COPD and evidence of AATD had a greater clinical burden as well as higher HCRU and spending compared with those with COPD alone (objective 2, cohort 2).

In a matched-cohort analysis, we compared individuals with COPD and evidence of AATD to individuals with COPD and no evidence of AATD. This real-world data analysis from the MAPD population demonstrated that individuals with COPD and AATD have a higher rate of pulmonary and hepatic comorbidities and exacerbations compared with individuals with COPD alone. Of note, patients with COPD and AATD had a greater pre-index comorbidity burden than patients with COPD but no AATD. This finding might be explained by the genetic nature of AATD, which predisposes individuals to comorbidities, including liver disease, not expected in non-AATD COPD.^19,20^ Importantly, hospitalizations due to various pulmonary events were higher in individuals with COPD and AATD than in those without AATD, even when baseline characteristics were risk-set matched.

A study by Sandhaus et al reported similar comorbidity trends among a Medicare 5% Fee-for-Service population; similarly, Greulich et al observed higher rates of comorbidities in individuals with COPD and AATD than in individuals with COPD alone among a German cohort.^18,21^

This high level of clinical burden in individuals with COPD and evidence of AATD was also reflected in higher COPD and pulmonary-event specific HCRU and spending among patients with COPD with evidence of AATD, compared with those without AATD. Specifically, all-cause healthcare spending at 12-month follow-up was almost twice as high in individuals with COPD and evidence of AATD compared with those without AATD. The greatest spending difference was observed for COPD-related medical spending, which was almost 4 times higher in individuals with COPD and AATD than in individuals with COPD alone. These results support findings in the literature indicating that individuals with COPD and AATD have greater HCRU and spending than individuals with COPD and no AATD.^13,21^

AATD testing rates among patients with newly diagnosed COPD were low (objective 3, cohort 3).

We further investigated the AATD testing rate among individuals who were newly diagnosed with COPD as seen in prior literature.^4,7,10,14^ In the MAPD population observed in this study, testing rates trended slightly upward from 2015 to 2020, but remained extremely low at about 1.5%. This result exposes the gap between real-world testing rates and guidelines, which recommend testing 100% of patients with COPD.^7^

Among patients with COPD, younger age and greater medical complexity were associated with increased rates of AATD testing. At the time of a COPD diagnosis, women and individuals living in suburban areas were more likely to be tested for AATD than men and individuals living in urban or rural areas. Further, White individuals were also more likely to be tested for AATD than Black individuals, which aligns with previous reports showing that AATD is more prevalent in White Americans than Hispanic and Black Americans.^22^

Previous studies reported similar results, although it is noteworthy that AATD testing rates in our study are lower than rates reported by Soriano et al in 2018 (~1.5% vs 2.2%).^10,14^ This might be explained by the higher average age or the predominantly White race of the MAPD population studied here. Specifically, AATD diagnosis after 60 years of age is less frequent compared with younger ages,^14^ and our results showed that White patients were more likely to be tested for AATD than Black patients. To further understand how insurance status may influence testing rates, studies of AATD testing rates in Medicare vs commercial insurance are of interest. One study by Sandhaus et al found that a commercially insured cohort contained more individuals with COPD and AATD than a Medicare cohort, although they did not report AATD testing rates.^18^

Delays in AATD diagnosis could be associated with greater disease severity and higher mortality.^11,23^ Therefore, broader testing for AATD among individuals diagnosed with COPD and their asymptomatic or presymptomatic family members, in line with evidence-based treatment guidelines, may aid in early identification and management of AATD. Awareness of their disease would allow both clinical and preclinical patients to adopt lifestyle modifications and receive appropriate care from an AATD rare disease specialist, possibly delaying or avoiding progression to severe disease.^2,4^ Specifically, smoking cessation, limited alcohol intake, physical exercise, and respiratory therapy are recommended to avoid proteolytic damage in individuals with AATD.^2^

Limitations common to studies using administrative claims data apply to this study. These include lack of specific clinical information such as certain billing codes, incomplete and missing data in the database, and errors in claims coding, including the possibility that individuals heterozygous for AATD (eg, Pi*MZ) were coded as AATD. No causal inference can be ascertained with this observational study using retrospective claims and chart data. While the Humana Medicare Advantage members included in our study are from a large national health plan residing across geographic regions, results may not generalize to the overall US population. Further, patients in the control group may have undiagnosed AATD that was not captured in any claims, and only a few individuals with AATD had available laboratory data. There is also the possibility that patients with direct-to-consumer testing (eg, AlphaID kit), as well as free testing programs (Alpha-1 Coded Testing study by the Alpha-1 Foundation), may not be captured in the claims database.^24,25^

Overall, this study shows that individuals with COPD and AATD have higher rates of comorbidities, HCRU, and spending than those without AATD. Testing rates for AATD slightly increased between 2015 and 2020 but remained low, which highlights the contrast between low testing rates and high clinical burden in individuals with AATD. Further research in a larger population is needed to assess the possible reasons for low AATD testing rates and the impact of improved AATD testing and treatment strategies on clinical outcomes, HCRU, and spending in those with COPD.

### Author Contributions

All authors are responsible for all content of the manuscript. All authors provided important intellectual input, critically reviewed and approved the work, and take responsibility for the final version of the manuscript for publication.

### Data Availability

The authors confirm that the data supporting the findings of this study are available within the article and its supplementary materials.

### Disclosures

N.K., J.H., and M.S. are employees of Takeda Pharmaceuticals USA, Inc., and are Takeda shareholders. S.H. was an employee of Takeda Pharmaceuticals USA, Inc. at the time of this study, and is a Takeda shareholder. T.N. was an employee of Takeda Pharmaceuticals USA, Inc. at the time of this study. A.D. is an employee of Humana Healthcare Research, Inc. B.T.S. and Y.X. are employees of Humana Healthcare Research, Inc. and are Humana shareholders. J.M.W. is an employee of the University of Alabama at Birmingham, received research support/grants from NIH/NHLBI, ARCUS-Med, Mereo BioPharma, Medscape, Verona Pharma, Grifols, Alpha-1 Foundation, Inhibrx, Department of Veterans Affairs, has patents issued or pending with Mereo BioPharma, has participated on Data Safety Monitoring Board or Advisory Board for AstraZeneca, Takeda, GSK, Bavarian Nordic, has stock options with Alveolus Bio, and has received equipment, materials, drugs, medical writing, gifts, or other services from Takeda, GSK, and Verona Pharma.

## Supplementary Material

Online Supplementary Material
