# Supplementary material for: Clinical and Economic Outcomes in Patients With Alpha-1 Antitrypsin Deficiency in a US Medicare Advantage Population
Source: J Health Econ Outcomes Res. 2025 Feb 20;12(1):66–74. doi: 10.36469/001c.127446 (PMC11846658; doi:10.36469/001c.127446)
Supplement: Online Supplementary Material [file jheor_2025_12_1_127446_266971.pdf]

Journal of Health Economics and Outcomes Research

Online Supplementary Material

Clinical and Economic Outcomes in Patients With Alpha-1 Antitrypsin Deficiency in a US Medicare Advantage Population. *JHEOR*. 2025;12(1):66-74. doi:10.36469/jheor.2025.127446

Table S1: Study Variable Descriptions

Table S2: P Values for Comparing Baseline Demographic and Clinical Characteristics Among Individuals With AATD, Severe AATD, and Without Evidence of Severe AATD

Table S3: P Values for Comparing Baseline Covariates Among Individuals With COPD and No AATD, and With Those With COPD and AATD

Table S4: P Values for Comparing Post-index HCRU in Individuals With COPD and With or Without AATD

Table S5: P Values for Comparing Baseline Demographic and Clinical Characteristics Among Individuals With Newly Diagnosed COPD Tested for AATD and Not Tested for AATD

Table S6: Baseline Demographics and Clinical Characteristics of Individuals Diagnosed With COPD and Without Evidence of AATD

Table S7: Pulmonary Events Associated With Hospitalization Among Individuals Diagnosed With COPD and Without Evidence of AATD

Table S8: All-Cause and COPD-Related Healthcare Spending Among Individuals Diagnosed With COPD, With and Without Evidence of AATD

Figure S1: Matched-Cohort Analysis Study Design

Figure S2: Pre-index Clinical Characteristics of Individuals Diagnosed With AATD, Overall, and Stratified by AATD Severity

Figure S3: Clinical Characteristics of Individuals Diagnosed With COPD With and Without Evidence of AATD

This is an open-access article distributed under the terms of the Creative Commons Attribution 4.0 International License (CCBY-4.0). View this license's legal deed at http://creativecommons.org/licenses/by/4.0 and legal code at http://creativecommons.org/licenses/by/4.0/legalcode for more information.

Figure S4: Alpha-1 Antitrypsin Deficiency Testing by Index Year

Figure S5: Comparative Clinical Characteristics of Individuals Newly Diagnosed With COPD

This supplementary material has been provided by the authors to give readers additional information about their work.

Table S1. Study Variable Descriptions

Variable	Description
AATD treatment patterns	**AATD treatment patterns** were examined during the follow-up period (12 months post-index) among individuals with a diagnosis of AATD.<br>Assessed **AATD treatments** as identified by medical and pharmacy claims were Glassia, Aralast NP, Prolastin-C, and Zemaira, which are all intravenously administered infusions.<br>**Number of treatment administrations** were measured as the number of medical and/or pharmacy claims on unique service dates with a code indicating an AATD treatment.<br>**Treatment duration** was measured as the number of days from first observed claim for an AATD treatment to the last observed claim during an AATD treatment.<br>**Treatment switch** was defined as a patient receiving a specific augmentation therapy and discontinuing that therapy, followed by having evidence of treatment with a different type of augmentation therapy.<br>**Treatment was considered discontinued** when patients had a treatment gap of ≥30 days.
AATD severity	**Severe AATD** was defined as:<br>• Laboratory AAT values were <50 mg/dL (as measured by nephelometry) anytime during the study period, *or*<br>• Patients with an AATD diagnosis and baseline hospitalizations for specific clinical conditions such as COPD exacerbations, respiratory distress, and respiratory failure in the 12-month pre-index period, *or*<br>• Patients with an AATD diagnosis and the use of supplemental oxygen in the 12-month pre-index period, *or*<br>• Patients with a diagnosis of AATD who received augmentation therapy medication in the 12-month pre-index or post-index period<br>**Moderate AATD** was defined as laboratory AATD values ≥50 mg/dL and ≤100 mg/dL
AATD testing in patients with newly diagnosed COPD	Testing for AATD was determined based on the presence of CPT codes 82103, 82104, 81332 on medical claims. To capture AATD testing that may occur before or after a COPD diagnosis, testing was assessed during the 12-month pre-index and 12-month post-index periods.
AATD-related health outcomes	**Pulmonary events or procedures** including chronic bronchitis, bronchiectasis, emphysema, exacerbations of COPD, lung transplant, pulmonary circulatory disorder, pneumothorax, adult-onset and late-onset asthma, and tuberculosis were examined. Conditions and procedures were identified based on the presence of diagnosis codes and procedure codes.<br>**Extrapulmonary manifestations** including panniculitis, vasculitis, aneurysms, fibromuscular dysplasia, granulomatosis with polyangiitis, eosinophilia, cirrhosis, chronic hepatitis, hepatocellular carcinoma, erythematous nodules, arterial hypertension, congestive heart failure, chronic kidney disease, and diabetes were examined. Conditions and procedures were identified based on the presence of diagnosis codes and procedure codes.<br>**Pulmonary events associated with hospitalization** were identified as conditions with diagnosis codes observed in the principal diagnosis position on an inpatient facility claims during an inpatient hospital episode. As a sensitivity analysis, more sensitive case definitions were also used that defined pulmonary events associated with hospitalization based on diagnosis codes present in any position on the inpatient facility claim.

Abbreviations: AAT, alpha-1 antitrypsin; AATD, alpha-1 antitrypsin deficiency; BETOS, Berenson-Eggers Type of Service; COPD, chronic obstructive pulmonary disease; CPI, Consumer Price Index; CPT, Current Procedural Terminology; ED, emergency department; HCPCS, Healthcare Common Procedure Coding System.

(Cont'd on page S4)

Table S1. Study Variable Descriptions

Variable	Description
Healthcare resource use	The number of **physician encounters** were all measured using Humana Healthcare Research, Inc (HHR) standard programs. Physician encounters were classified by CPT/HCPCS and BETOS categorization.
	**Inpatient episodes of care** were derived from facility claims using bill type and revenue codes as well as dates of service. Multiple inpatient episodes where the discharge and the admit dates are within one day (reflecting a transfer) were collapsed into a single inpatient episode. ED encounters that result in an inpatient admission were considered part of the subsequent inpatient admission.
	**ED encounters** were identified using revenue codes, place of treatment codes, and CPT/HCPCS codes.
	**COPD-specific inpatient hospitalizations and ED visits** were identified based on medical claims with primary diagnosis of COPD or exacerbation.
	**Pulmonary events** related utilizations and healthcare spending were calculated based on medical claims with primary diagnosis of the following conditions: lung transplant, pulmonary circulatory disorder, tuberculosis, panniculitis, vasculitis, aneurysms, fibromuscular dysplasia, granulomatosis with polyangiitis, eosinophilia, adult-onset asthma.
Healthcare spending	**All-cause total healthcare spending** were calculated using HHR standard programs. Payment level is derived from source of billing (facility or professional) and place of service (physician office or facility). Spending for all services were adjusted to 2020 US dollars utilizing the Medical Consumer Price Index (CPI).
	**COPD-specific** healthcare spending was identified based on medical claims with primary diagnosis of COPD or exacerbation.

Abbreviations: AAT, alpha-1 antitrypsin; AATD, alpha-1 antitrypsin deficiency; BETOS, Berenson-Eggers Type of Service; COPD, chronic obstructive pulmonary disease; CPI, Consumer Price Index; CPT, Current Procedural Terminology; ED, emergency department; HCPCS, Healthcare Common Procedure Coding System.

Table S2. *P* Values for Comparing Baseline Demographic and Clinical Characteristics Among Individuals With AATD, Severe AATD, and Without Evidence of Severe AATD

Measure	*P* Value[a]
Mean age	<.001
Sex	.917
Race	.257
Geographic region	.540
Population density	.412
LIS	.005
DE	.244
LIS or DE	.007
Median Deyo-Charlson Comorbidity Index	<.001
Median Elixhauser Comorbidity Index	<.001

Abbreviations: AATD, alpha-1 antitrypsin deficiency; DE, dual eligible; LIS, low-income subsidy.
[a]*P* value was calculated using χ^2 test for categorical variables and Wilcoxon rank sum test for continuous variables.

Table S3. *P* Values for Comparing Baseline Covariates Among Individuals With COPD and No AATD, and Those With COPD and AATD

Measures	*P* Value[a]
Conditions, n (%)	
Bronchitis (not specific)	.020
Bronchitis (chronic + not specific)	.024
Bronchiectasis	<.001
Emphysema	<.001
Adult-onset asthma	<.001
Events, n (%)	
Exacerbations of COPD	<.001
Pneumothorax	.001
Procedures, n (%)	
Lung transplant	<.001
Conditions, n (%)	
Bronchitis (not specific)	.049
Bronchitis (chronic + not specific)	.074
Bronchiectasis	<.001
Emphysema	<.001
Adult-onset asthma	<.001
Exacerbations of COPD	<.001
Pneumothorax	.086
Procedures, n (%)	
Lung transplant	<.001
Cirrhosis	<.001
Diabetes	<.001
Eosinophilia	.077
Hepatocellular carcinoma	.073
Cirrhosis	<.001
Diabetes	<.001
Eosinophilia	.007
Hepatocellular carcinoma	.004

Abbreviations: AATD, alpha-1 antitrypsin deficiency; COPD, chronic obstructive pulmonary disease.
[a]*P* value was calculated using χ^2 test for categorical variables and Wilcoxon rank sum test for continuous variables.

Table S4. *P* Values for Comparing Post-index HCRU in Individuals With COPD and With or Without AATD

**Measures[a] (n)**	***P* Value[b]**
Inpatient admissions	
All-cause	
No. of patients with an IP visit	.011
Median length of stay	.643
COPD-specific[c]	
No. of patients with an IP visit	<.001
Median length of stay	.230
Pulmonary event–specific[d]	
No. of patients with an IP visit	.804
Median length of stay	.325
ED visits	
All-cause	
No. of patients	.521
Median ED visits	.168
COPD-specific[c]	
No. of patients	<.001
Median ED visits	.795
Pulmonary event–specific[d]	
No. of patients	.002
Median ED visits	.983
Physician office visits	
All-cause	
No. of patients	.032
Median office visits	<.001

Abbreviations: AATD, alpha-1 antitrypsin deficiency; COPD, chronic obstructive pulmonary disease; ED, emergency department; HCRU, healthcare resource utilization; IP, inpatient.
[a]Spending and HCRU are measured using data from the 12-month post-index period.
[b]*P* value was calculated using χ^2 test for categorical variables and Wilcoxon rank sum test for continuous variables.
[c]COPD-specific inpatient and ED visits were identified based on medical claims with a primary diagnosis of COPD or exacerbation.
[d]Pulmonary event-related utilizations were calculated based on medical claims with primary diagnosis of the following conditions: lung transplant, pulmonary circulatory disorder, tuberculosis, panniculitis, vasculitis, aneurysms, fibromuscular dysplasia, granulomatosis with polyangiitis, eosinophilia, and adult-onset asthma.

Table S5. *P* Values for Comparing Baseline Demographic and Clinical Characteristics Among Individuals With Newly Diagnosed COPD Tested for AATD and Not Tested for AATD

Measures	*P* Value[a]
Median age	<.001
Sex	<.001
Race	<.001
Geographic region	.259
Population density	.056
LIS, n (%)	.519
DE, n (%)	.886
LIS or DE	.592
Median Deyo-Charlson Comorbidity Index	<.001
Median Elixhauser Comorbidity Index	<.001

Abbreviations: AATD, alpha-1 antitrypsin deficiency; COPD, chronic obstructive pulmonary disease; DE, dual eligible; LIS, low-income subsidy.
[a]*P* value was calculated using χ^2 test for categorical variables and Wilcoxon rank sum test for continuous variables.

Table S6. Baseline Demographics and Clinical Characteristics of Individuals Diagnosed With COPD and Without Evidence of AATD

Measures	Patients With COPD Without AATD,[a] (n = 7420)	Patients With COPD With AATD,[b] (n = 742)	Standardized Difference	*P* Value[c]
Age, y				
Mean (SD)	68 (9)	68 (9)	0	>.999
Median (IQR)	69 (61–75)	69 (61–75)		
Sex, n (%)				
Men	3340 (45.0)	334 (45.0)	0	>.999
Women	4080 (55.0)	408 (55.0)	0	
Race, n (%)				
White	7210 (97.2)	721 (97.2)	0	>.999
Black	210 (2.8)	21 (2.8)	0	
Geographic region, n (%)				
Northeast	226 (3.0)	18 (2.4)	0.037	.333
Midwest	1435 (19.3)	157 (21.2)	0.047	
South	5061 (68.2)	489 (65.9)	0.049	
West	698 (9.4)	78 (10.5)	0.037	
Population density, n (%)				
Urban	3974 (54.4)	385 (52.3)	0.042	.021
Suburban	2211 (30.3)	257 (34.9)	0.098	
Rural	1052 (14.4)	92 (12.5)	0.056	
Unknown	62 (0.8)	<10	0.068	
LIS, n (%)	2076 (28.0)	186 (25.1)	0.066	.0912
DE, n (%)	732 (9.9)	75 (10.1)	0.007	.833
LIS or DE	2100 (28.3)	188 (25.3)	0.068	.086
Deyo-Charlson Comorbidity Index				
Mean (SD)	2.5 (2.1)	2.3 (1.8)	0.11	
Median (IQR)	2 (1–4)	2 (1–3)		.066

(Cont'd on page S8)

Measures	Patients With COPD Without AATD[a] (n = 7420)	Patients With COPD With AATD[b] (n = 742)	Standardized Difference	*P* Value[c]
Deyo-Charlson Comorbidity Index, category				
0	828 (11.2)	45 (6.1)	0.18	<.001
1	2206 (29.7)	296 (39.9)	0.22	
2	1532 (20.6)	138 (18.6)	0.05	
3	996 (13.4)	118 (15.9)	0.07	
4+	1858 (25.0)	145 (19.5)	0.13	
Elixhauser Comorbidity Index				
Mean (SD)	4.2 (2.9)	4.0 (2.6)	0.06	
Median (IQR)	4 (2–6)	3 (2–5)		.277
Elixhauser comorbidities, n (%)				
Congestive heart failure	1327 (17.9)	113 (15.2)	0.07	.071
Cardiac arrhythmia	1483 (20.0)	153 (20.6)	0.01	.681
Valvular disease	633 (8.5)	67 (9.0)	0.02	.644
Pulmonary circulation disorder	417 (5.6)	53 (7.1)	0.06	.090
Peripheral vascular disease	1489 (20.1)	113 (15.2)	0.13	.002
Hypertension, uncomplicated	5014 (67.6)	487 (65.6)	0.04	.282
Hypertension, complicated	1074 (14.5)	94 (12.7)	0.05	.180
Paralysis	83 (1.1)	<10	0.08	.069
Other neurological disorder	499 (6.7)	45 (6.1)	0.02	.492
Chronic pulmonary disease	5696 (76.8)	663 (89.4)	0.34	<.001
Diabetes mellitus, uncomplicated	2045 (27.6)	159 (21.4)	0.14	<.001
Diabetes mellitus, complicated	1352 (18.2)	90 (12.1)	0.17	<.001
Hypothyroidism	1298 (17.5)	146 (19.7)	0.06	.137
Renal failure	1172 (15.8)	102 (13.7)	0.06	.143
Liver failure	337 (4.5)	48 (6.5)	0.09	.018
Peptic ulcer disease, no bleed	78 (1.1)	<10	0.02	.783
HIV/AIDS	22 (0.3)	0 (0.0)	0.08	.138
Lymphoma	57 (0.8)	<10	0.04	.490
Metastatic cancer	83 (1.1)	<10	0.03	.438
Solid tumor, no metastasis	576 (7.8)	51 (6.9)	0.03	.386
Rheumatoid arthritis, collagen disease	508 (6.8)	43 (5.8)	0.04	.277
Coagulopathy	274 (3.7)	20 (2.7)	0.06	.165
Obesity	1207 (16.3)	97 (13.1)	0.09	.024
Weight loss	282 (3.8)	43 (5.8)	0.09	.008
Fluid electrolyte disorder	965 (13.0)	115 (15.5)	0.07	.056
Blood loss anemia	86 (1.2)	<10	0.08	.123
Deficiency anemia	383 (5.2)	45 (6.1%)	0.04	.293
Alcohol abuse	226 (3.0)	12 (1.)	0.09	.027
Drug abuse	443 (6.0)	28 (3.8)	0.1	.014
Psychoses	157 (2.1)	12 (1.6)	0.04	.363
Depression	1616 (21.8)	152 (20.5)	0.03	.415
Anxiety	2155 (29.0)	214 (28.8)	0	.908

Abbreviations: AATD, alpha-1 antitrypsin deficiency; COPD, chronic obstructive pulmonary disease; DE, dual eligible; IQR, interquartile range; LIS, low-income subsidy; SD, standard deviation.

[a]Controls were identified and matched to COPD with AATD patients within each calendar year independently. Control patients already matched in previous years are not eligible for matching in later years.

[b]This is the AATD cohort identified in objective 1 with the following modifications: (1) required COPD during 12-month pre-AATD index date; (2) excluded patients with unknown race or race other than White or Black.

[c]*P* value was calculated using χ^2 test for categorical variables and Wilcoxon rank sum test for continuous variables.

Table S7. Pulmonary Events Associated With Hospitalization Among Individuals Diagnosed With COPD and Without Evidence of AATD

Measures	Patients With COPD Without AATD Diagnosis	Patients With COPD With AATD Diagnosis	*P* Value[a]
Hospitalizations for pulmonary comorbidities (principal diagnosis position), n (%)			
Bronchiectasis	<10	<10	<.001
Emphysema	11 (.1)	<10	.108
Exacerbations of COPD	253 (3.4)	47 (6.3)	<.001
Hospitalizations for pulmonary comorbidities (any diagnosis position), n (%)			
Bronchiectasis	15 (.2%)	10 (1.3)	<.001
Emphysema	98 (1.3)	24 (3.2)	<.001
Exacerbations of COPD	653 (8.8)	111 (15.0)	<.001

Abbreviations: AATD, alpha-1 antitrypsin deficiency; COPD, chronic obstructive pulmonary disease.
[a] *P* value was calculated using χ^2 test for categorical variables and Wilcoxon rank sum test for continuous variables.

Table S8. All-Cause and COPD-Related Healthcare Spending Among Individuals Diagnosed With COPD, With and Without Evidence of AATD

Measures	Individuals With COPD Without AATD	Individuals With COPD With AATD	*P* Value[a]
Total all-cause spending ($), median (IQR)	$11 113 (4956-26 671)	$19 375 (9178-50 722)	<.001
Medical spending, median (IQR)	$6230 (2455-19 063)	$8492 (3864-22 962)	<.001
COPD-specific[b] medical spending, median (IQR)	$219 (0-900)	$815 (277-2288)	<.001
Pharmacy spending, median (IQR)	$2521 (812–5845)	$5564 (2151–13 141)	<.001

Abbreviations: AATD, alpha-1 antitrypsin deficiency; COPD, chronic obstructive pulmonary disease; IQR, interquartile range.
[a] *P* value was calculated using χ^2 test for categorical variables and Wilcoxon rank sum test for continuous variables.

Figure S1. Matched-Cohort Analysis Study Design

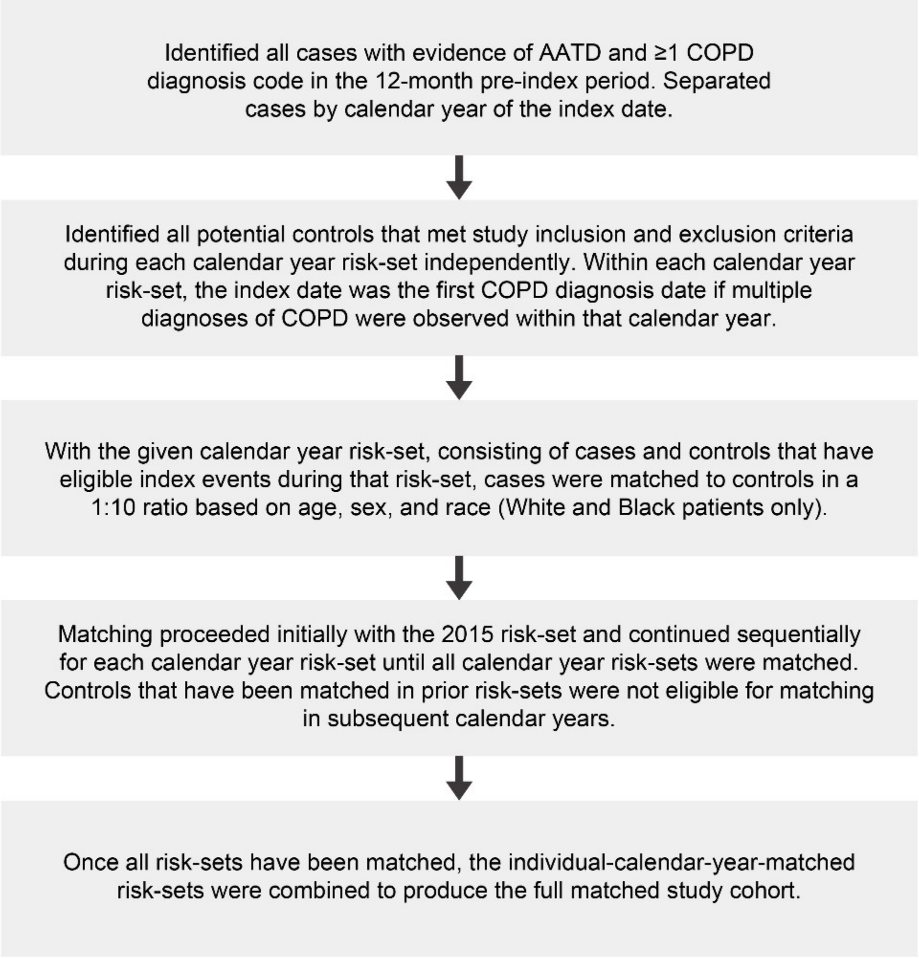

Abbreviations: AATD, alpha-1 antitrypsin deficiency; COPD, chronic obstructive pulmonary disease.

Figure S2. Pre-index Clinical Characteristics of Individuals Diagnosed With AATD, Overall, and Stratified by AATD Severity

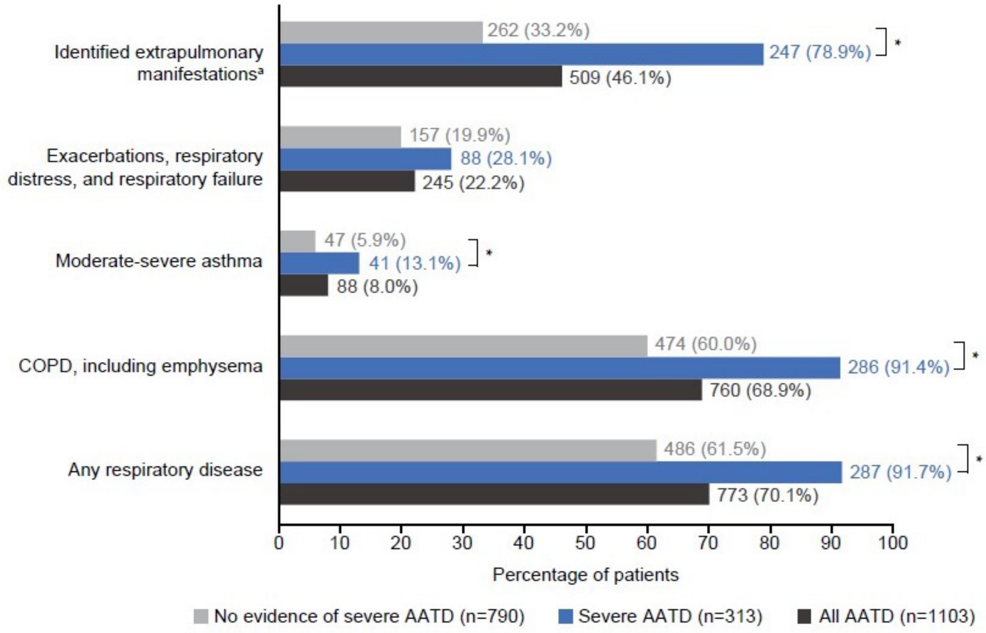

Abbreviations: AATD, alpha-1 antitrypsin deficiency; COPD, chronic obstructive pulmonary disease.
[a]Includes panniculitis, chronic hepatitis, chronic kidney disease, and congestive heart failure.
**P* < .001.

Figure S3. Clinical Characteristics of Individuals Diagnosed With COPD With and Without Evidence of AATD

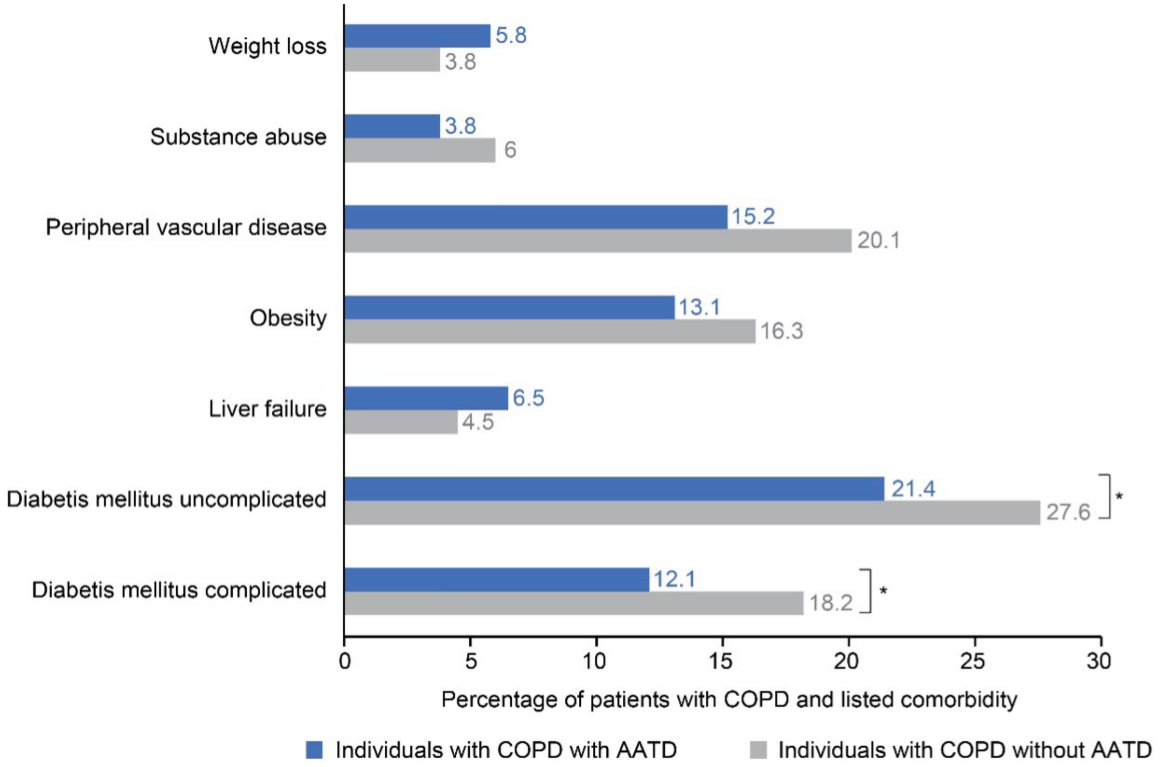

Abbreviations: AATD, alpha-1 antitrypsin deficiency; COPD, chronic obstructive pulmonary disease.
*$P < .001$.

Figure S4. AATD Testing by Index Year

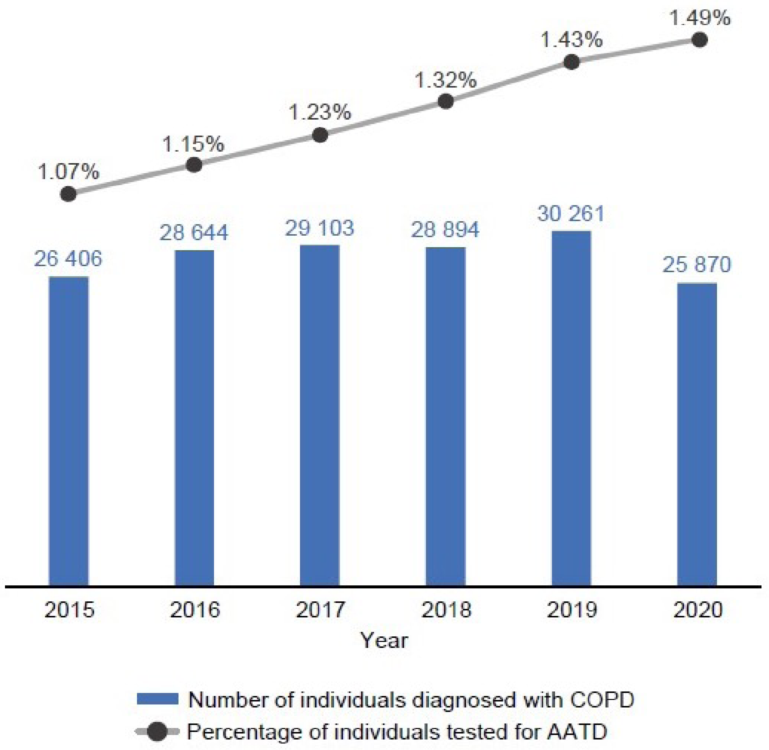

Abbreviations: AATD, alpha-1 antitrypsin deficiency; COPD, chronic obstructive pulmonary disease.

Figure S5. Comparative Clinical Characteristics of Individuals Newly Diagnosed With COPD

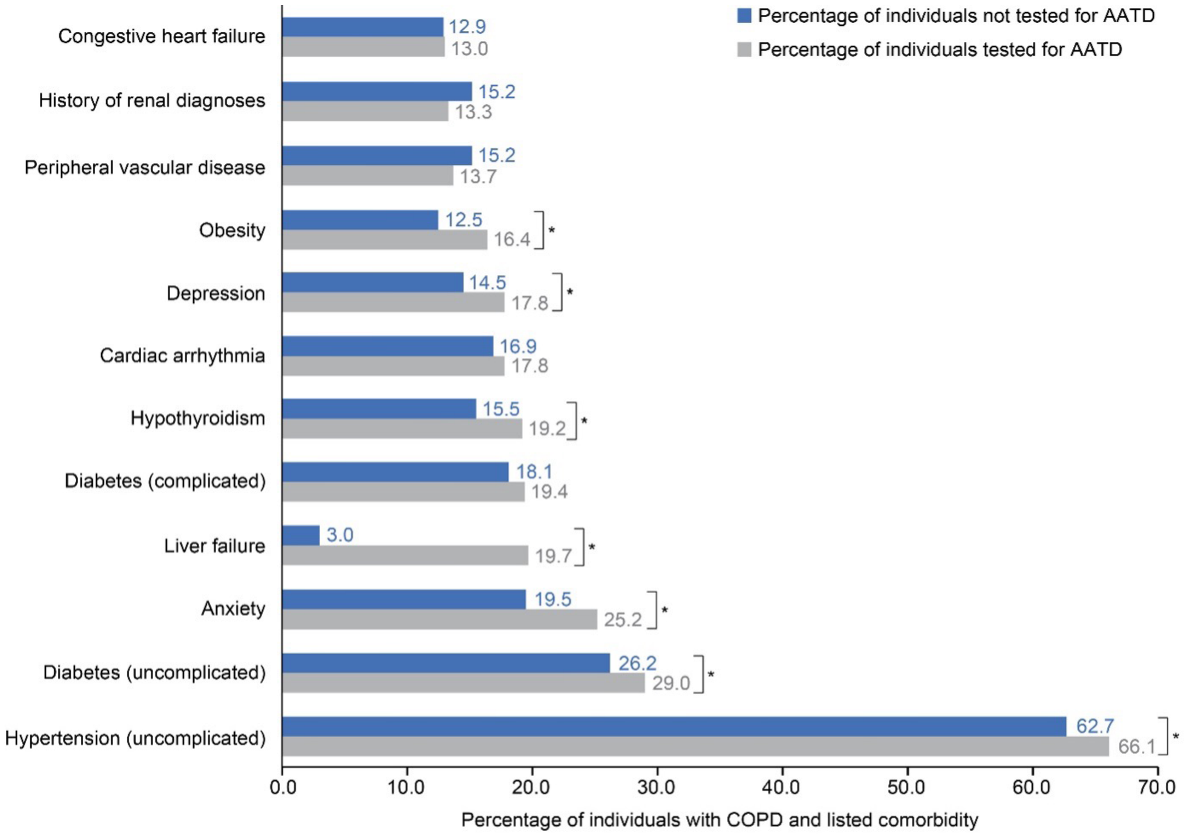

Abbreviations: AATD, alpha-1 antitrypsin deficiency; COPD, chronic obstructive pulmonary disease.
*$P < .001$.
